# Psychological Aspect and Quality of Life in Porphyrias: A Review

**DOI:** 10.3390/diagnostics12051193

**Published:** 2022-05-10

**Authors:** Francesca Granata, Annamaria Nicolli, Alessia Colaiocco, Elena Di Pierro, Giovanna Graziadei

**Affiliations:** 1 Dipartimento di Medicina Interna, Fondazione IRCCS Cà Granda Ospedale Maggiore Policlinico, 20122 Milan, Italy; elena.dipierro@unimi.it (E.D.P.); giovanna_graziadei@yahoo.it (G.G.); 2 Department of Cardiac, Thoracic, Vascular Sciences and Public Health, University of Padova, 35128 Padova, Italy; annamaria.nicolli@unipd.it; 3 Dipartimento di Psicologia, IUSTO Istituto Universitario Salesiano Torino Rebaudengo, 10155 Torino, Italy; alessiacolaiocco@gmail.com

**Keywords:** porphyrias, psychological aspect, QoL, pain, stigma, biopsychosocial approach

## Abstract

The World Health Organization (WHO) describes “health” as a state of physical, mental, and social well-being and not merely the absence of disease or infirmity. Therefore, a biopsychosocial approach should be considered as an integral part of patients’ management. In this review, we summarize the available data starting from 1986 on the biological, psychological, and social aspects of porphyrias in order to provide a useful tool for clinicians about the missing knowledge within this field. Porphyrias are a group of rare metabolic disorders affecting the heme biosynthetic pathway and can be categorized into hepatic and erythropoietic. Here, a total of 20 articles reporting the psychological and the quality of life (QoL) data of porphyria patients affected by acute hepatic porphyrias (AHPs), Porphyria Cutanea Tarda (PCT), and Erythropoietic Protoporphyria (EPP) were analyzed. These 13 articles include reported quantitative methods using questionnaires, while the reaming articles employed qualitative descriptive approaches through direct interviews with patients by psychology professionals. We conclude that the use of questionnaires limits the complete description of all areas of a patient’s life compared to a direct interview with specialists. However, only a combined use of these methods could be the best approach for the correct disorder management.

## 1. Introduction

Porphyrias are a group of nine rare genetic metabolic disorders caused by alterations in the enzymes involved in the heme biosynthetic pathway [1]. Porphyrias are classified as either *hepatic* or *erythropoietic*, depending on the principal accumulation site of the toxic precursors of heme synthesis such as 5-aminolevulinic acid (ALA), porphobilinogen (PBG), and porphyrins [2].

Hepatic porphyrias include delta-aminolevulinic acid dehydratase-deficiency porphyria (ADP; OMIM 612740), acute intermittent porphyria (AIP; OMIM 176000), hereditary coproporphyria (HCP; OMIM 121300), variegate porphyria (VP; OMIM 176200), and porphyria cutanea tarda (PCT; OMIM 176100), whereas the erythropoietic porphyria group consists of X-linked protoporphyria (XLP; OMIM 300752), congenital erythropoietic porphyria (CEP; OMIM 263700), hepatoerythropoietic porphyria (HEP; OMIM 176100), and erythropoietic protoporphyria (EPP; OMIM 177000) [3,4,5]. Several variations in the prevalence of porphyrias have been observed due to its rare occurrence that ranges from only a few cases of ADP and CEP, described worldwide to less than 1:1500 for AIP [6,7]. The prevalence of EPP ranges from 1:75000 to 1:150000 [8]. Patients usually present with either neurotoxic or phototoxic symptoms depending on the type of porphyrin precursor that accumulates in the body [1,7,9].

Among the hepatic porphyrias (AHPs), AIP, HCP, and VP are characterized by neurological attacks that typically exhibit abdominal pain, nausea, vomiting, tachycardia, hypertension, anxiety, agitation, paralysis, and respiratory arrest. However, all symptoms are not concomitantly present in every neurological exacerbation [10,11]. These acute attacks often begin after puberty, are more common in females, and are usually triggered by different medications, calorie restriction, infections, surgery, stressful situations, and various hormonal changes [12,13,14]. The HCP and VP forms show not only acute neurovisceral symptoms but also photosensitivity due to the accumulation of photoreactive porphyrins [5,15].

Despite PCT being classified as hepatic, it is characterized by chronic symptoms similar to erythropoietic porphyrias, such as photosensitivity, skin fragility, and blistering on sun-exposed areas. It typically develops in late adulthood and exists as two clinical subtypes: familial, due to inherited genetic defect, and a sporadic subtype, which is acquired [16,17].

The erythropoietic porphyrias XLP and EPP are characterized by phototoxic symptoms triggered by visible light range at 410 nm. The occurrence of symptoms usually arises within a few minutes of skin exposure, with burning and itching that, after several hours of exposure, leads to severe symptoms such as erythema, swelling, edema, blistering, as well as, in some cases, purpura [18]. Repeated phototoxic episodes might result in an altered skin appearance with permanent cutaneous manifestations. Moreover, 20–30% of patients have a liver implication, and 5% need liver transplantation [8,19,20,21,22,23,24,25].

CEP is the most severe form of erythropoietic porphyrias characterized by bullous cutaneous photosensitivity to visible light from early infancy, progressive photo mutilation, and chronic hemolytic anemia [4].

Low quality of life (QoL) characterizes all forms of porphyria despite different symptoms. Frequent acute attacks in AHPs have a highly variable course, lasting from several months to many years, and they affect the health of patients throughout their lives [26]. The onset of erythropoietic porphyrias symptoms start from early childhood, resulting in light avoidance behaviour, thus limiting their social, educational, and professional activities throughout the year as well as leading to feelings of social isolation [27,28,29].

Although several studies have described the clinical and pathophysiological characteristics of porphyrias, little is known about their correlation with the psychological aspect and with social life. With an increase in clinical studies for the therapeutic assessment and management of porphyrias, these aspects have received more attention in recent years.

**Figure 1:**
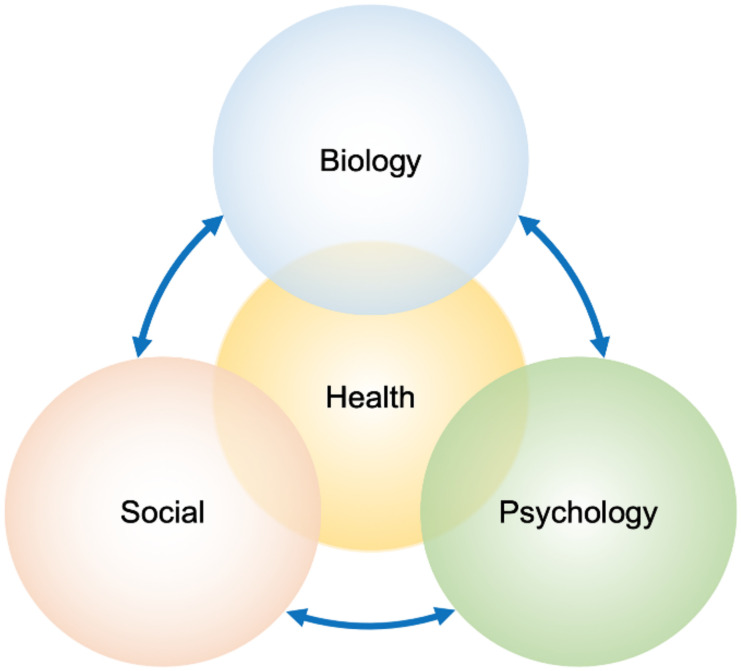
The key characteristics of the biopsychosocial approach with three different aspects during patient care: the biological component of the disease as symptoms, the psychological impact of the disease, and the patient’s social context. Every component in a chronic disorder can affect the patient’s health.

In this review, for the first time, we summarized 20 articles on AIP, PCT, and EPP about the biopsychosocial aspects (Figure 1) [30,31,32]. In the following paragraphs and tables, we summarized the assessment method, the age of the population, the number of patients, the number of items or hours of interview, and the significant score resulting from the questionnaires.

## 2. Methods

We started our work assuming the absence of reviews in the literature that summed up all published articles on the psychological aspect and QoL in porphyrias. The search strategy was conducted to identify studies that examined the pool of selected Medical Subject Headings (MeSH^®^) words in this field. We conducted a detailed literature search in English to identify all studies performed from 1986 to date. The full electronic search strategy used was “porphyria” restricted to either MeSH^®^ major topic/“Emtree term” of the article (PubMed and EMBASE, respectively) AND Psychology, QoL.

Studies that met the following criteria were included: (i) an original study published in a peer-review journal; (ii) studies conducted in the last 35 years; (iii) all age groups were considered; (iv) all cohort dimensions were considered due to the rarity of the pathologies.

Using these MeSH words and criteria, only 20 articles emerged from the literature, and all were considered. These articles were analyzed by considering the methodologies used in the studies, and thus distinguishing between the questionnaires (13 articles) and qualitative approaches (7 articles) employed through direct interviews with patients by psychology professionals. To summarize the articles, we divided them according to the classification of porphyrias (10 of 20 articles describe the AHPs; 4 on PCT; and 6 on EPP).

Moreover, other MeSH words were selected on the biopsychosocial approach; chronic disorder; chronic pain; rare disorder always combined with the other two MeSH used before psychology and QoL. We selected another 48 articles to perform comparisons on the management of other chronic conditions/rare disorders at the psychological and social levels or to discuss the biopsychosocial approach, focusing the attention on the importance of all these aspects (biological, psychological, and social) in the management of the diseases. The other 32 articles focused on porphyrias, and were used to describe the disorders at the clinical level.

## 3. Acute Hepatic Porphyrias (AHPs)

### 3.1. Questionnaire Assessment of Psychological Aspect of AHPs

Millward et al. proposed the first study in 2001, in which four different self-reported questionnaires were used for assessing 81 patients (AIP: 51, VP: 25, HCP: 5; Table 1). The results for the Medical Outcomes Study (MOS) questionnaire showed an inferior health status to porphyria patients when compared to the normal population. Moreover, the EuroQoL Questionnaire (EuroQoL) showed marked differences in QoL between porphyria patients and normal population in males aged 70 to 79 years and in females aged 60 to 69 years (1.00, with no remarkable issues). The Hospital Anxiety and Depression Scale (HADS) analysis also displayed higher anxiety levels than depression in the porphyria-affected population. While the Illness Perceptions Questionnaire (IPQ) reported that porphyria did not affect the physical, social, and psychological functioning as severely as diabetes, in fact, for this questionnaire, the author used a diabetic patient cohort to compare porphyria patients (Table 1) [11].

Because it was extremely important to elucidate the contrasting IPQ results of AHP patients versus the diabetic cohort, the analysis was performed again using three different groups of patients as follows: (A) group-asymptomatic patients with a latent disease showing no signs of porphyria; (S) group-symptomatic patients who had single or a few acute attacks once in the lifetime; and (R) group-patients categorized as recurrent cases with >4 attacks per year that required frequent hemin infusion for four consecutive days or frequent hospitalization (Figure 1).

With this approach, the results of latent S patients could be compared with non-porphyritic patients, whereas the R patients showed higher depression and anxiety scores on the HADS scale despite having a lower score on the Medical Outcomes Study (MOS) index. Moreover, the IPQ score assessment resulted in significant differences between the perceived consequences of porphyria, with the R group reporting higher health distress ratings than those with latent symptomology [11].

In 2005, the same author applied other questionnaires to 90 patients (58 AIP, 32 VP), demonstrating that the mental health status of patients with porphyria is an important feature of the porphyria patient experience. The data revealed higher levels of anxiety than depression on both BAI and HADS measures, suggesting that anxiety appears to be more prominent than depression. With regard to the comparison of scores between manifest and latent cases, raised anxiety was higher in manifest patients; however, there were no statistically significant differences in the nature of this anxiety. This suggests that the nature of raised anxiety in the porphyria population cannot be explained by manifest symptomology [33]. The anxiety status, depression, and other negative emotions are well described in chronic illness patients with health-related QoL in chronic diseases that might interfere in all aspects of patients’ lives, including education, work, and social life [34,35,36].

**Figure 2:**
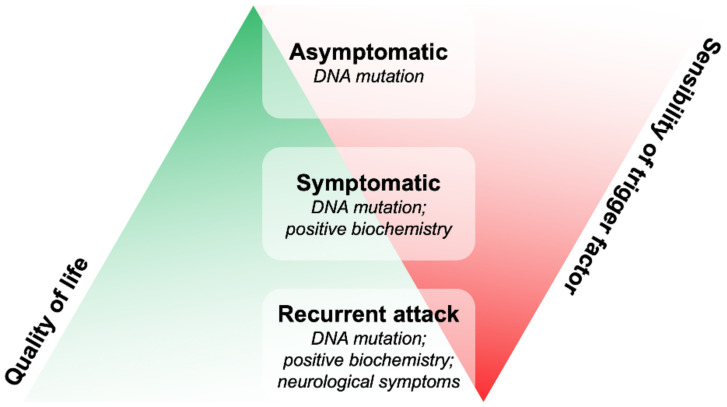
AHP symptoms are divided into groups of onset: (R) recurrent attack, (S) symptomatic, and (A) asymptomatic patients. The green triangle represents the patients’ quality of life, which increases at the apex. The red one represents the sensitivity to triggering factors that increase at the top of the pyramid.

Over the years, several other indices such as the Katz Index of Independence in Activities of Daily Living (Katz-ADL) and the Barthel Index (BI) have been utilized in AHP patients for assessing activities of daily living (ADLs). Based on this, the results displayed that 81.25% of the porphyric group was relatively independent, whereas 6.25% showed a lower level of dependency, and 12.5% exhibited moderate dependency in contrast to the control group that showed total independence. The evaluation of QoL using the EuroQol five-dimensional (EQ-5D) questionnaire displayed significant differences on the visual analog scale (VAS) and the pain perception indicator while depicting lower values in the AIP group than in the control group (Figure 2, Table 1) [37].

A 2018 study conducted for 27 women from 25 Chinese families exhibited that AIP patients had significant QoL differences when compared to the general population, as demonstrated by the self-reported SF-36 questionnaire (85.74 vs. 91.83, *p* = 0.001). In addition, post-traumatic stress disorder (PTSD) measurements have also been computed by the Impact of Event Scale-Revised questionnaire (IES-R). It was observed that the patients were afraid and nervous of intermittent attacks, whereas AIP patients reported an increased prevalence of PTSD-related symptoms along with the finding that patients without an acute attack (A group) had better IES-R in the intrusion score than those who had acute attacks (R group). Moreover, distress and anxiety have been a constant lingering issue due to the inadequacy of the treatment strategies, as hematin is not used therapeutically to treat acute porphyria in China (Table 1) [38].

A German study on a cohort of 62 patients (57 AIP, 5 VP), composed of 84% females, using the QoL questionnaire, reported that more than 70% of the patients had some limitations regarding their spare time activities. Traveling long distances was especially difficult or even impossible due to paralysis cases, fear of acute attacks, fatigue, or weakness and a constant requirement for medical care. Due to these reasons, the patients were assigned an average of 5 with a range of 0–8 for the degree of impairment of their general QoL on a scale from 0 to 10 (Table 1) [39].

In the last two studies, some new aspects emerged for AHP patients, as PTSD and the chronic fatigue are not only related to the acute attack. In fact, the fatigue is an emerging characteristic in many different chronic illnesses, such as autoimmune, rare disorder, and liver diseases [40,41].

The introduction of long-term, new innovative therapy by Alnylam Pharmaceuticals led to a global study on 112 AHP patients called “EXPLORE”. This study was used a custom Porphyria Patient Experience Questionnaire (PPEQ), consisting of eight questions assessing the different aspects of patients’ lives, out of which five questions emerged from the EuroQoL 5-dimensions questionnaire 5-levels [EQ-5D-5L] that were specific for the patient’s QoL. The study depicted EQ-5D-5L Index Scores as 0.78 in the AIP population, with a median age of 38 years, which was lower than the normal European population of 0.92 (for the 35–44 years of age group). In addition, a principal aspect that emerged from this study was the constant presence of chronic symptoms that adversely influenced the day-to-day functioning of AHP patients [42].

### 3.2. Qualitative Approach of the Psychological Aspect of AHPs

The first qualitative analysis conducted by Wikberg et al. in 2000 involved the direct interviewing of five women coping with an aggressive form of AIP (Table 1). The results revealed severe physical pain and psychological experiences, followed by different mood swings, apprehension of having seizures, as well as an innate fear of being considered as an individual with a mental illness or addicted to morphine used to relieve unbearable pain during acute attacks [12].

A second qualitative study was conducted in 2016 on 16 patients (15 AIP; 1 VP) using a focus group method, which enumerated the patients’ prodromal symptoms occurring days before an attack, such as a state of brain fog accompanied by disorientation, irritability, fatigue, anxiety, and agitation, without any abdominal pain [43]. Another study assessing 19 AIP patients, including 15 females with 2-h qualitative one-on-one interviews with a semi-structured guide, reported that AIP caused a profound impact on several areas of their lives, namely alterations in sleep pattern as well as the ability to work, walking abnormalities, dwindling finances with increased medical costs, and decreased socialization [44].

A recent analysis by the British Porphyria Association involved optional 1-h telephonic interviews of both patients and caregivers while demonstrating that pain was the most frequently reported acute and chronic symptom for AHP patients, requiring further therapeutic management. Although a majority of patients had experienced an acute attack, 94% of them experienced chronic symptoms between those acute attacks that adversely affected daily living and the QoL [45]. A thorough literature review of porphyria since 2000 suggests that acute porphyria is not only an “intermittent” disorder, but also depicts long-standing chronic implications, several of which are debilitating and negatively affect the QoL. Moreover, it results in a new perspective, as it appears to have acute exacerbations as well as chronic manifestations, leading to limitations in a patient’s ability to function regularly while having remarkable social, psychological, and biological adverse consequences.

## 4. Porphyria Cutanea Tarda (PCT)

**Figure 3:**
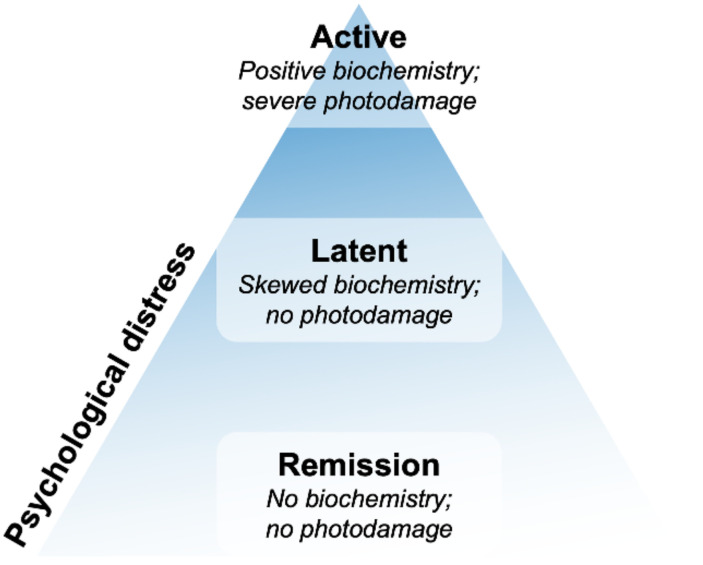
PCT symptoms are divided into three groups of onset: (R) remission, (L) latent, and (A) asymptomatic. The triangle represents the patients’ psychological distress, which increases at the apex. The base of the pyramid represents the major involvement of psychological distress.

The limited available research regarding the psychosocial impact of PCT disease led to a study by Jong et al. in 2008 that concluded that photodermatoses of PCT majorly impacted the QoL. However, PCT had a lesser impact on QoL than other cutaneous porphyrias, probably due to the late onset of the disease [46]. In this study, results were attributed to a limited sample size (12 patients), having patients in the remission period, along with minor psychological distress (Figure 3) [22]. These results are in contrast with the 2015 qualitative data collected by analyzing three 90-min interviews, based on three main themes: experience with symptoms, treatment, and the future expectations of 21 patients (11 women; 10 men) (Table 2). Data showed a large variation in experiences, from mild to extremely dramatic symptoms, with a greater psychosocial and QoL impact on the affected individuals.

The above-mentioned data were confirmed by a 2016 analysis by the same research group involving 263 patients screened through three different questionnaires, namely, Brief Illness Perception Questionnaire (BIPQ), Subjective Health Complaints (SHC), and Impact of Events Scale [IES], that accurately measured psychological distress (Table 2).

The patients were categorized into three groups, as show in Figure 3: (R) remission (172/263), (L) latent (34/263), and (A) active (57/263), depending on their persistence of symptoms [47]. The results showed a strong discrepancy in the scores obtained in the R and L categories, whereas the A group displayed higher scores in all the applicable questionnaires (Figure 2, Table 2). Thus, it was stated that PCT was a chronic systemic debilitating disease with recurrent and vigorous symptoms, followed by the effectual reporting of more health complaints by active PCT individuals. Active PCT patients perceived PCT as involving more discomfort and psychological involvement when compared to patients with L and R PCT. However, in these groups, the experienced psychological distress indicated a possible new onset of symptoms [48].

Another study evaluating 61 patients for the impact of PCT on QoL between December 2013 and December 2015 [46,49] used the 12-item Short-Form Health Survey–Version 2 (SF-12v2), which was a generic measure of health aspects. It included, firstly, the Physical Component Summary (PCS), whose low scores indicated the limitations in physical functioning, a higher degree of body pain, as well as poor general health and, secondly, the Mental Component Summary (MCS), which reflected frequent psychological distress in lower scores.

Although the general mean value was similar to the results of the general population, an analysis of different aspects of these subgroups indicated that a few symptoms, such as itching, were associated with the reduced QoL (Table 2).

Similar to AIP, the above-stated results indicated the importance of selecting the right cohort of patients affected with the same disease stage to have an accurate distress evaluation in the different stages of the disease, which further paved the way for specific psychological interventions promoting social and emotional development in accordance with the R, L, and A disease phases.

Emotion regulation strategies in chronically ill patients play a crucial role in proper disease management because higher emotional intelligence contributes to better psychological health and enhanced physical benefits due to a lower risk of depression and anxiety disorder, as an appropriate emotional regulatory behavior involves recognizing and dealing with the varied negative appearances in different stages of the disorder [50,51]. On the contrary, inefficient emotional health control measures can adversely affect a patient’s health, leading to an evasion of protective health behaviors as well as non-adherence to the pertinent treatment [52].

## 5. Erythropoietic Protoporphyria (EPP)

### 5.1. Questionnaire Assessment of Psychological Aspect of EPP

In the last few years, the introduction of a new, effective drug for EPP treatment, afamelanotide, developed by Clinuvel Pharmaceuticals, made it easier to monitor the QoL enhancement in the absence of a blood marker for an accurate assessment of the improvement in a patient’s photosensitivity [28,53,54,55].

Based on this, a clinical trial including 74 patients from Europe and 94 patients from the United States assessed the QoL using the EPP disease-specific Quality of Life (EPPQoL) questionnaire. This described the QoL consequences in two domains, general well-being as well as the impact of the disease severity, and showed a significantly increased QoL (expressed as the percentage of the maximum (100% quality)) over the baseline value (74% vs. 31%; Table 2) after drug administration [55].

Similarly, an Italo-Swiss study evaluating the positive effects of afamelanotide on 173 patients (120 Italian and 53 Swiss) by the EPP-specific QoL questionnaire, using the Likert scale on behavior [56], reported a lower QoL score (4 out of 10) in EPP adults when compared to the control group (Table 2). Moreover, it was discovered that the QoL measurement in adolescents was 2.6, as compared to 4, the score for adults, which might be due to a proper alignment of daily activities and routine to the reported disease limitations in adult patients (Table 3) [57].

The former results were in broad agreement with the available past literature for managing adolescent chronic disorders because adolescents with recurrent pain conditions reported poorer attendance, increased academic pressure, lower school satisfaction, and a greater likelihood of physical bullying due to the constant interference of chronic pain in the students’ academic performance and school attendance [58,59,60].

Further evidence of a correlation between EPP and poor QoL has been reported in 39 Swiss patients using a specific EPP questionnaire that revealed a lower percentage of QoL (Table 3) when compared to the maximum score of 100% before starting the treatment with afamelanotide [27]. The low QoL was predominantly due to the pain perception, as described in several other chronic diseases [61,62,63], thus reinforcing the inverse correlation of chronic pain and the QoL, as shown in (Figure 1) [51,64,65].

Naik et al., in 2019, conducted two studies on the biopsychosocial aspect of 202 US patients using different questionnaires distributed unequally among all patients due to certain protocol modifications after initiating the studies. The Hospital Anxiety and Depression Scale (HADS) results showed that 20% of the patients had borderline anxiety, followed by depression in 10% of subjects, which was similar to the percentage of depression and anxiety in the normal US population.

Although the Illness Perception Questionnaire-Revised (IPQ-R) index measures an individual’s beliefs and feelings about an impending illness, a higher score was obtained, depicting the negative impact of EPP/XLP on a patient’s life, in particular, with the consequences linked to the disease and the chronicity of symptoms (Table 2).

The results were confirmed by the most robust approach, the Patient-Reported Outcomes Measurement Information System 57 (PROMIS-57) questionnaire, which analyzed (1) biological aspects as pain interference, fatigue perception, and physical function; (2) psychological aspects such as anxiety, depression, and sleep disturbances; (3) social aspects, including the assessments of life satisfaction and aspects of daily life. Several PROMIS domains had significantly different scores, specifically for the correlation between the pain interference, physical function, fatigue, and satisfaction with the social impact while highlighting the most relevant aspect as the pain interference in the relevant aspects of a person’s daily life, including photosensitivity reactions [66,67,68,69].

### 5.2. The Qualitative Approach of the Psychological Aspect of EPP

Rufener et al. conducted a study on the psychological impact of EPP in 1986 using a standardized written questionnaire containing 25 questions, followed by a structured 3–6-h interview (Table 3), on a 12-subject cohort comprising three females (37.3 ± 8.08 years) and seven males (29.71 ± 7.08 years), including a girl (7 years) and a boy (11 years). The study brought to light other parameters such as the invisibility of symptoms and lack of pain credibility from others that affected the correct diagnosis. In terms of the psychological aspect, the major impact observed in patients was burning and a pain sensation as patients felt detached from their surroundings and often found it difficult to sleep, followed by an increase in suicidal thoughts as well as expressing the fear of death by five individuals, who thought that overexposure to sunlight could prove fatal. Henceforth, it can be concluded that individuals afflicted with EPP suffer from a tremendous psychological burden that can have devastating outcomes due to self-limiting beliefs, as highlighted in this review characterizing EPP. In general, every chronic disorder, in the absence of evident tissue damage due to invisible symptoms, can lead to extreme difficulty in communicating the disease and its severity to others [70,71].

Because the physical pain expressed by patients is disproportionate to the lesion’s extent, it results in the observation that these affected individuals suffer from psychosomatic diseases or hypochondriasis [72] that must be acknowledged in detail along with an array of invisible symptoms to provide substantial evidence that individuals with chronic disorders are commonly stigmatized [73]. Pain-related stigma was also identified as a public health priority in the 2016 National Pain Strategy (NPS) that delineated a coordinated plan for reducing the burden of chronic pain and its potential impact on delayed diagnosis, treatment bias, and impairment in treatment recovery [58]. This was also in accordance with Lippe et al.’s study in 2017 that described that over half of the former studies reported substantial negative impacts as well as patients’ perceptions of stigma and social misconception due to the presence of a rare chronic disorder [74] along with the innate fear of being categorized negatively [60].

In 2019, a qualitative study was performed for studying the psychological aspect through open discussion focus groups, without using questionnaires, for children and young adults with the parents’ assessment (Table 3), whereas the questions were designed on the following biopsychosocial aspects: (1) feelings about EPP, (2) social life, (3) experience with phototoxic episodes, and (4) family and home life. The obtained results revealed a negative general impact of EPP on the biopsychosocial aspect of the patients’ as well as parents’ lives. This was further proved by the fact that the children expressed frustration, sadness, and jealousy due to the inability to participate in frequent daytime school activities, whereas the young adults reported that middle school and high school days were the most difficult times while dealing with EPP and its limitations [75].

## 6. Discussion

Considering the importance of introducing the biopsychosocial approach in the care of patients with a chronic disorder, this review wanted to provide an overview regarding the psychological impact and the resultant QoL involvement of porphyrias, a group of rare chronic heme metabolism disorders [76]. We attempted to summarize all 20 original articles of the last 35 years regarding this topic, underling the differences between results obtained by quantitative or qualitative approaches.

In Table 1, Table 2 and Table 3, we have summed up data reported in all analyzed articles such as age; the number of patients involved in the studies; the number of items (if are questionnaires) or hours of interviews; and the more impactful score obtained from the questionnaire. In total, the articles screened 482 AHP, of which were 287 AIP, 72 VP, 9 HCP, 308 PCT, and 596 EPP patients over 18 years. Only two articles involved adolescence and childhood for 24 EPP patients aged 7 to 11 years [71,75]. The results show that data about psychological and QoL aspects on porphyria mainly refer to the adult population, suggesting a missing analysis at the adolescent and child age.

Moreover, we noted that the number of studies on psychological and QoL aspects on porphyrias has increased significantly in the past decades, showing an increasing number of assessments conducted using both self-completion questionnaires and direct interviews with relevant subject experts.

We noticed that the generic questionnaires were less accurate, with the risk of minimizing the disease’s severity. It is known that the qualitative research method utilizing semi-structured, direct interviews is the best psychological approach that analyses the dynamic interaction between the three essential health aspects of patients based on the biopsychosocial model: the biological component of the disease as symptoms, the psychological impact of the disease, as well as the patient’s social context [64,77,78] (Figure 1).

However, some articles have reported modified questionnaires specific for porphyria that show more precise results, such as in the study on AHPs by Yang et al., which highlighted an additional symptom of post-traumatic stress, or those emerging in the EXPLORE study of chronic symptoms that influenced the day-to-day functioning of AHP patients [38,42].

The issue of a questionnaire’s sensitivity is a well-known scientific concern that can influence both the obtained data and any additional evidence. The non-sensitivity of the questionnaire might lead to different disease perceptions between the asymptomatic, symptomatic, and remission groups, as reported in AHP patients studies [33,79], or in the use of the QoL questionnaire for PCT, which was non-specific and modeled on other dermatological disorders [46]. On the contrary, the use of qualitative studies, based on the direct approach with patients, achieved satisfactory results on the definition of the psychological impact of the disease, providing new therapeutic insights about AHP and PCT (Figure 2 and Figure 3) [1,39,80,81].

Moreover, in the qualitative studies, both hepatic and erythropoietic porphyria analyses showed the implication of several areas of a patient’s life involved in the disorder’s management [44]. Between those, the underlying fear of an inability to heal properly from the acute phase, the fear of dying [12], and repeated suicidal thoughts [71], followed by the additional findings that patients’ social lives were restricted in the different age groups over the years due to a strong impact on their personal and social behaviors. Moreover, EPP-affected individuals fear a social stigma due to the invisibility of their symptoms and may even lie about his or her state of health for fear of being classified as different or socially isolated [71].

A psychological involvement due to the chronicity of the disease and the symptoms’ invisibility has also been reported in AHPs with several other emerging aspects common in all chronic diseases, such as mood swings, coping strategies, fatigue, sleep quality, and pain interference, on daily activities [32,64,77,82,83,84,85]. For EPP, the literature is not completely exhaustive to state the chronicity of disease, even if new evidence of it has been described since the Clinuvel clinical trials [55,57].

The major symptoms emerging in AIP and EPP patient questionnaires are depression and anxiety, which must be taken into consideration in the clinical management of patients as a consequence of chronic symptoms due to porphyrias impacting in daily QoL. The psychological discomfort, due to the chronic disease, is an aspect largely described as leading to the lowering of the quality of care and negative patient outcome if not treated and taken into consideration in a patient’s management [86,87].

All Qol results for AHP are characteristic of a chronic disorder that can induce profound changes in a person’s life, not only during the acute attack, but also in daily life, with a lot of efforts from patients being required to manage chronic symptoms such as fatigue, malaise, and pain [38,39,88,89,90,91].

For EPP-affected individuals, the reported QoL impact was low, not only due to the phototoxic events, but also due to the daily routine activity management along with improper day-to-day planning in the absence of the required therapy [92].

Due to the limited research into the interaction between PCT and its impact on QoL, we can conclude that a lower QoL was associated only with the presence of symptoms in the active group. However, the distress in latent and recurrent patients was described as being linked to the possibility of a new onset of symptoms [48].

Over the past few decades, the recent emerging trends to diagnose, treat, and manage AHPs have resulted in defining them as a chronic and not only intermittent disease triggered by certain predisposing factors [76,82]. With regard to EPP, a wider health perspective should be developed to consolidate the fact that it is a chronic disease and not just a seasonal one [92,93,94].

## 7. Conclusions

In conclusion, all data reported underline the chronicity of all type of porphyrias that affect the psychological and QoL aspects of a patient’s life, as happens for other chronic disorders [95,96,97,98], introducing a new vision compared to the past classification that saw the porphyrias divided into acute and chronic. All this evidence emerged through the patient’s assessment with both specific questionnaires designed and validated for porphyrias and through direct approaches. In fact, during the counselling, the patients were free to express all discomfort that the disease causes, not only during the symptoms, highlighting that a major source of anxiety and depression is due to the management of daily life to avoid the symptoms.

Furthermore, the importance of introducing psychologic assessment into the multidisciplinary approach, which the porphyrias have at a clinical level, could be useful to understand new pathophysiological aspects of the disease and also to help the patient and caregivers in the management of daily life in the presence of these chronic disorders.

## Figures and Tables

**Table 1:** The sum-up of literature of AHPs: author; methodology; age; number of screened patients; number of the item; significative score; the maximum score. NR = not reported.

Authors	Assessment Method	Age (Years)	Patients (n)	Item (n)	Score	Score Max
Quantitative methods by using questionnaires						
Millward LM, et al., 2001	Medical Outcomes Study (MOS)	>18	81 AIP: 51 VP: 25 HCP 5	20 item	Health Perception 54.1 (±2.4)	100
	EuroQoL Questionnaire (EuroQoL)			NR	M 70–79 yrs = 0.10 F 60–69 yrs = 0.63	1
	Hospital Anxiety and Depression Scale (HADS)			14 item	Anxiety 7.1 ± 4.9 Depression 4.6 ± 4.7	21
	Illness Perceptions Questionnaire (IPQ)			NR	NR	NR
Millward LM, et al., 2005	Beck Anxiety Inventory (BAI)	>18	90 58 AIP 32 VP	21 item	10.3 ± 9.4	63
	Beck Depression Inventory (BDI)			21 item	8.5 ± 8.5	63
	State Trait Anxiety Inventory (STAI)			20 item		80
	Hospital Anxiety and Depression Scale (HADS)			14 item	NR	21
	General Health Questionnaire (GHQ12)			12 item	2.21 ± 3.42	1
Jiménez-Monreal AM, et al., 2015	Health-related Quality of Life Questionnaire (EQ-5D)	>18	32 (AIP)	5 domains	VAS 61.60	100
	Activities of Daily Living (Katz-ADL and Barthal Index)			10 item	81.5% independent	100
Yang J, et al., 2018	SF-36 Health Survey (Chinese version 1.0)	mean 29	27	36 item	85.74	100
	Event Scale-Revised Questionnaire (IES-R)			22 item	36.7 ± 11.8	> 26
Bronisch O, et al., 2019	Porphyria-oriented Quality of Life Questionnaire	>18	62	9 item	5	10
Gouya L, et al., 2020	EuroQoL 5–Dimensions Questionnaire 5–Levels (EQ–5D–5L)	≥18	112 104 AIP 3 HCP 5 VP	5 item	0.78	1
Qualitative descriptive approaches						
Wikberg A, et al., 2000	Qualitative approach (interwiew)	mean 55	5	20 ± 45 min	NR	NR
Naik H, et al., 2016	Focus group with interactive discussion	>18	16 15 AIP 1 VP	1.5–2 h interview	No score	NR
Simon A, et al., 2018	Qualitative one-on-one interviews	mean 40	19	2 h interview	No score	NR
Gill L, et al., 2021	Online survey	≥18	38 28 AIP 9 VP 1 HCP	NR	No score	NR
	Telephone interview		10 on 38 were eligible	1 h interview		

**Table 2:** The sum-up of the literature of AHPs: author of publications; methodology approach used for the psychological or QoL assessment; age; number of screened patients; number of the item; field; significative score; the maximum score. NR = not reported.

Authors	Assessment Method	Age (Years)	Patients (n)	Item (n)	Score	Score Max
Quantitative methods by using questionnaires						
Jong CT, et al., 2008	Dermatology Life Questionnaire Index (DLQI)	Mean 57	12	10 item	>10	30
Andersen J, et al., 2016	Brief Illness Perception Questionnaire (BIPQ)	25–78	263	8 item	A group 39.6 (35.8–43.4) R group 29.7 (27.9–31.5) L group 27.2 (23.3–31.1)	Between group *p* < 0.01
	Self-reported Health Complaints (SHC)			29 item	A group 20.5 (16.7–24.2) R group 14.1 (12.4–15.8) L group 10.7 (7.7–13.7)	
	INTRUSION: Impact of Events Scale (IES)			7/15 item	A group 11.7 (9.1–14.3) R group 6.1 (5.1–7.1) L group 3.0 (1.6–4.4)	
	AVOIDANCE: Impact of Events Scale (IES)			8/15 item	A group 12.1 (9.5–14.7) R group 6.1 (5.1–7.1) L group 4.3 (2.2–6.4)	
Andersen J, et al., 2020	Short Form-12 Health Survey vs. 2 (SF-12)	24–79	12	12 item	PCS mean 48 MCS mean 47	Mean 50
Qualitative descriptive approaches						
Andersen J, et al., 2015	Qualitative approach (interactive discussion)	31–77	21	1.5 h of interview	Higher impact of disease	NR

**Table 3:** The sum-up of the literature of AHPs: author of publications; methodology approach used for the psychological or QoL assessment; age; number of screened patients; number of the item; field; significative score; the maximum score. NR = not reported.

Authors	Assessment Method	Age (Years)	Patients (n)	Item (n)	Score	Score Max
Quantitative methods by using questionnaires						
Langendonk JG, et al., 2015	EPP disease-specific Quality of Life Questionnaire (EPPQoL)	>18	167	12-item	31%	100%
Biolcati G, et al., 2015	EPP—specific Quality of Life (QoL) Questionnaire by Clinuvel	>18	173	18/16-item	4	10
Naik H, et al., 2019 Apr.	PROMIS	>18	193	NR	Higher for pain	NR
	HADS		103	NR	Border line	NR
	IPQR		104	NR	Higher	>20
	XLP/EPP-specific tools		107	7 day recall	Higher	100
Barman-Aksözen J, et al., 2020	EPP-QoL Questionnaire	>18	35	12 item	49.10%	100%
Qualitative descriptive approaches						
Rufener AE. 1989	EPP Questionnaire + Structurated interview	>18	10	25-item	NR	NR
	Structurated interview	7–11 >18	12	3–6 h of interview		
Naik H, et al., 2019 Jan.	Qualitative approach (focus group with interactive discussion)	10-nov	6	17 open questions	NR	NR
		24–25	4			
		Parents 29–55	14			
